# A decouple-decomposition noise analysis model for closed-loop mode-localized tilt sensors

**DOI:** 10.1038/s41378-023-00614-z

**Published:** 2023-12-21

**Authors:** Kunfeng Wang, XingYin Xiong, Zheng Wang, Liangbo Ma, BoWen Wang, WuHao Yang, Xiaorui Bie, ZhiTian Li, XuDong Zou

**Affiliations:** 1 The State Key Laboratory of Transducer Technology, Aerospace Information Research Institute, Chinese Academy of Sciences, Beijing, China; 2 School of Electronic, Electrical and Communication Engineering, University of Chinese Academy of Sciences, Beijing, China; 3 Ming Hsieh Department of Electrical and Computer Engineering, University of Southern California, Los Angeles, CA USA; 4 Shangdong Key Laboratory of Low-altitude Airspace Surveillance Network Technology, QiLu Aerospace Information Research Institute, Jinan, China

**Keywords:** Electrical and electronic engineering, Sensors

## Abstract

The development of mode-localized sensors based on amplitude output metrics has attracted increasing attention in recent years due to the potential of such sensors for high sensitivity and resolution. Mode-localization phenomena leverage the interaction between multiple coupled resonant modes to achieve enhanced performance, providing a promising solution to overcome the limitations of traditional sensing technologies. Amplitude noise plays a key role in determining the resolution of mode-localized sensors, as the output metric is derived from the measured AR (amplitude ratio) within the weakly coupled resonator system. However, the amplitude noise originating from the weakly coupled resonator’s closed-loop circuit has not yet been fully investigated. This paper presents a decouple-decomposition (DD) noise analysis model, which is applied to achieve high resolution in a mode-localized tilt sensor based on a weakly coupled resonator closed-loop circuit. The DD noise model separates the weakly coupled resonators using the decoupling method considering the nonlinearity of the resonators. By integrating the decoupled weakly coupled resonators, the model decomposes the weakly coupled resonator’s closed-loop circuit into distinct paths for amplitude and phase noise analyses. The DD noise model reveals noise effects at various circuit nodes and models the system noise in the closed-loop circuit of the weakly coupled resonators. MATLAB/Simulink simulations verify the model’s accuracy when compared to theoretical analysis. At the optimal operating point, the mode-localized tilt sensor achieves an input-referred instability of 3.91 × 10^-4°^ and an input-referred AR of PSD of 2.01 × 10^-4°^⁄√Hz using the closed-loop noise model. This model is also applicable to other varieties of mode-localized sensors.

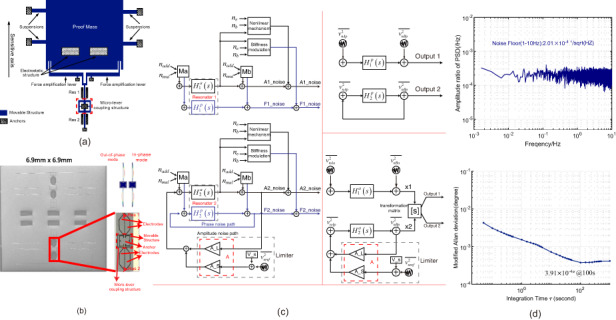

## Introduction

Micro/nanomechanical resonator sensing technology has substantially advanced as driven by the ever-growing comprehension and utilization of fundamental physical phenomena. Various intrinsic physical phenomena, such as internal resonance^1–4^, phase synchronization^5–7^, phonon-cavity^8–11^, and mode localization^12–14^, have been developed and employed to overcome the sensing resolution of the resonator(s). Among these nonlinear physics explorations, notable achievements include noise reduction and stability enhancement, along with quality factor improvement through internal resonance and phase synchronization^1^. Moreover, sensitivity enhancement has been realized through phonon-cavity and mode localization in both single or coupled micro/nanomechanical resonator systems^11,12^. Universally, these phenomena apply to oscillatory systems and impact the energy dynamics within resonators, affecting more than one resonator mode or vibrational element. The phonon-cavity method facilitates mode coupling, which can occur in single or coupled resonators, and it is compatible with both strong and weak coupling between modes of these resonators^10,15^. Moreover, mode localization can enhance sensing sensitivity due to the highly localized displacement realized through weak coupling phenomena.

Recently, a new generation of coupled resonator sensors, known as mode-localized sensors, has been developed by leveraging the concept of mode localization^12,13,16–18^. These sensors operate on the principle of mode localization, a phenomenon wherein the energy of the weakly coupled resonators becomes spatially confined within one of the resonators, thereby enhancing the sensitivity of the sensors based on the amplitude output metrics. In addition, these sensors have shown the ability to enhance the sensitivity of resonant sensors by two to four orders of magnitude when compared to traditional resonant sensors^14,16,19^. Mode-localized sensors also exhibit excellent common-mode rejection of environmental temperature and pressure variations due to their identical coupled resonators^20^. These properties make mode-localized sensors ideal for detecting various physical parameters, such as displacement^21^, charge^22,23^, acceleration^14,24,25^, and tilt^26–28^.

Tilt sensors are essential in diverse application fields, where both long-term stability and high sensitivity are crucial performance requirements. In construction and engineering, tilt sensors with such features ensure precise monitoring of structural inclination, detecting even the smallest deviations over extended periods. Automotive and transportation applications demand tilt sensors that combine long-term stability and high sensitivity to provide accurate levelling and suspension control for optimal vehicle performance and safety. In aerospace and aviation, where precise orientation is also critical, tilt sensors must exhibit both long-term stability and high sensitivity to enable accurate attitude determination and reliable flight control. Similarly, in robotics, tilt sensors with excellent long-term stability and high sensitivity contribute to precise posture control and navigation in dynamic environments. Across these varied fields, the aforementioned tilt sensors are required to ensure reliable and accurate angle measurements and high-quality performance. The application of mode localization to tilt sensors is a natural extension of this prior research, as these sensors require both high sensitivity and long-term stability to function effectively in various fields. Tilt sensors measure the inclination angle of an object relative to the gravitational force acting upon it, providing essential information for numerous control systems^29,30^. The inherent characteristics of mode-localized sensors, such as their amplified response to small perturbations and their inherent robustness against environmental temperature and pressure, make them highly suitable for tilt sensing applications^20^.

There has also recently been a strong emphasis on enhancing the sensitivity of mode-localized sensors through structure design^25,31^ or expanding the system’s Degrees of Freedom (DoF)^14,19^, resulting in improved signal detection and measurement capabilities. Furthermore, significant efforts have been devoted to optimizing noise performance, including noise analysis and closed-loop system optimization^32–35^, enabling mode-localized sensors to achieve higher precision and accuracy in various sensors.

Related studies on the optimal operating points for the different output metrics of the coupled resonators were previously shown for the special condition of amplitude ratio (AR) of approximately 1.23 and amplitude difference of approximately 0.23, i.e., near the veering point^26,32^. Hemin Zhang’s research suggests that the optimal operational amplitude approaches the critical amplitude, at which the sensor exhibits superior noise floor performance and enhanced stability^36^. However, it is necessary to use the weakly coupled resonator closed-loop circuit to track and stabilize the frequency and amplitudes of the mode-localized sensors. Therefore, further analysis of amplitude noise in mode-localization sensors based on weakly coupled resonator closed-loop circuits is of great significance. To address this issue, we propose a decouple-decomposition (DD) noise analysis model based on the weakly coupled resonator’s closed-loop circuits and specifically designed for the analysis and optimization of amplitude noise in mode-localized tilt sensors. The DD noise model separates the weakly coupled resonators using the decoupling method considering the nonlinearity of the resonators. By integrating the decoupled weakly coupled resonators, the model decomposes the weakly coupled resonators closed-loop circuit into paths for amplitude noise and phase noise analysis. This model decomposes noise effects at various circuit nodes, thereby analyzing the noise sources that adversely affect the overall sensor performance. A resulting expression for system noise in the weakly coupled resonator closed-loop circuit is established. MATLAB/Simulink simulations verify the model’s high accuracy compared with theoretical analysis. At the optimal operating point, the mode-localized tilt sensor achieves an input-referred instability of 3.91 × 10^−4^° and an input-referred AR of PSD of $2.01\times {10}^{-4{\rm{^\circ }}}/\sqrt{{Hz}}$. This model is also applicable to other varieties of mode-localized sensors, enabling high-resolution tilt sensors that utilize the mode-localization effect.

## Results and discussion

### Device structure and characterization

**Fig. 1:**
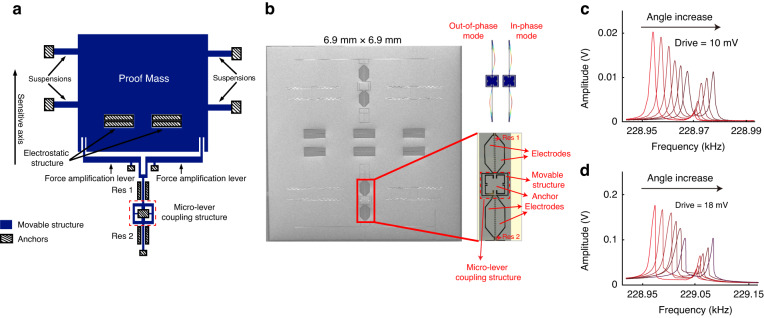
Device structure and characterization. **a** Schematic view of the mode-localized tilt sensor. **b** Optical image of the weakly coupled resonators and vibration modes of the weakly coupled resonators. Open-loop amplitude-frequency response curves of resonator 2, subjected to varying tilt angles, with driving voltages set at **c** 10 mV and **d** 18 mV

The mechanical configuration of the mode-localized tilt sensor comprises a proof mass supported by four suspensions, a pair of differential microlever force amplifiers, two identical clamped-clamped (C–C) resonators connected by a mechanical microlever coupling structure, and a virtual electrostatic tuning structure, as shown in Fig. 1a, b^25,26^. The dimensions of the mode-localized tilt sensor are provided in Supplementary Materials Table I. Upon the occurrence of a tilt variation, the proof mass exerts an inertial force, which is subsequently amplified by the microlever force amplifiers and applied to one of the coupled resonators (resonator 1). This axial inertial force alters the stiffness of the weakly coupled resonator system, resulting in the distribution of energy across the weakly coupled resonators. Consequently, the vibrational amplitudes of the two resonators significantly change, giving rise to the mode-localization effect between the weakly coupled resonators^12,16^. The mechanical microlever coupling structure connects the two identical resonators, leading to two coupled vibration modes, as shown in Fig. 1b: out-of-phase (mode 1) and in-phase (mode 2). The resonant frequency discrepancy between the two coupled modes is attributed to fabrication tolerance and can be rectified by employing the tuning structure^25^.

**Fig. 2:**
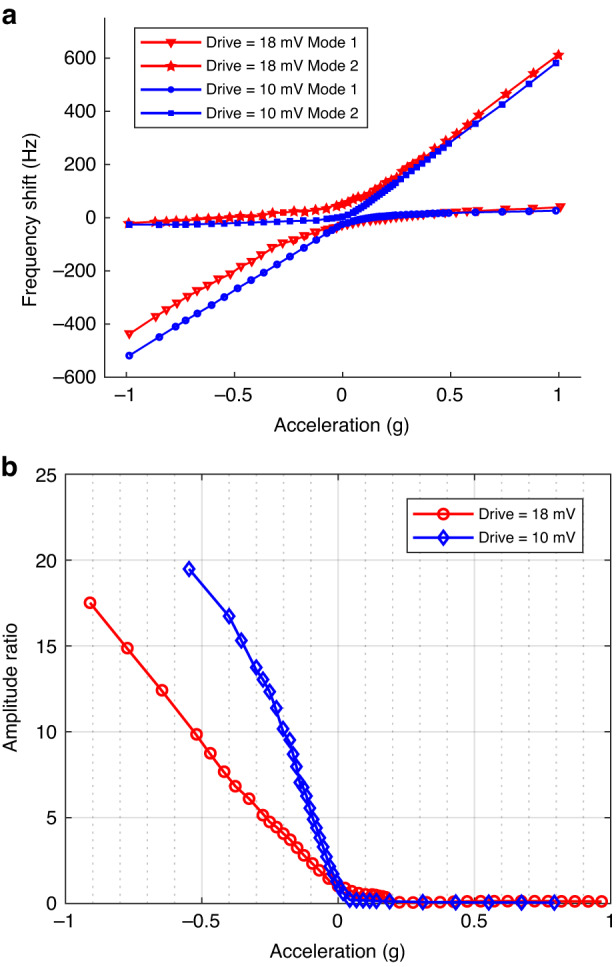
The closed-loop characterization. Acceleration plotted against frequency **a** and AR **b** at driving voltages of 10 mV and 18 mV

Figure 1c, d show the open-loop amplitude-frequency response curves of resonator 2 as subjected to varying tilt angles with driving voltages set at 10 mV and 18 mV, respectively. These curves show the alterations in the vibration amplitudes of the two coupled modes in response to different tilt angles. The frequency discrepancies between mode 1 and mode 2 are approximately 18.75 Hz and 46.76 Hz when the driving voltages are set at 10 mV and 18 mV, respectively. Additionally, with these driving voltages correspond to sensitivities of approximately 0.79 AR/° and 0.64 AR/° (Amplitude Ratio/°), respectively, as shown in Fig. 2. The experimental configuration is further described in Supplementary Material Fig. S1.

### Decoupling the weakly coupled resonators

Assuming that the two coupled resonators are identical (the effective mass and stiffness are *m*_1_ = *m*_2_ = *m* and ${k}_{1}={k}_{1}=k$, respectively), the stiffness of the coupling spring *k*_*c*_ is much less than the stiffness of the resonators *k* (*k*_*c*_ ≪ *k*). The dynamics equations of weakly coupled resonators can thus be given as

1a $${m}_{1}{\ddot{x}}_{1}+\left({k}_{1}+{k}_{c}\right){x}_{1}-{k}_{c}{x}_{2}=f(t)$$

1b $${m}_{2}{\ddot{x}}_{2}+\left({k}_{2}+{k}_{c}\right){x}_{2}-{k}_{c}{x}_{1}=0$$

where $f\left(t\right)=F\sin \left({\omega }_{d}t+\theta \right)$ is the driving force, ${\omega }_{d}$ is the drive force frequency, ${x}_{1}$ and ${x}_{2}$ are the displacements of resonator 1 and resonator 2. Converting physical coordinates $\{x\}$ to modal coordinates $\{y\}$ is achieved by

2 $$\left\{x\right\}=\left[s\right]\left\{y\right\}$$

Substituting (2) into (1) yields

3 $${\left[s\right]}^{T}\left[m\right]\left[s\right]\left\{\ddot{y}\right\}+{\left[s\right]}^{T}\left[k\right]\left[s\right]\left\{y\right\}={\left[s\right]}^{T}\left\{F(t)\right\}$$

$$\left[s\right]=\frac{1}{\sqrt{2}m}\left[\begin{array}{cc}1 & 1\\ 1 & -1\end{array}\right]$$ is the transformation matrix. Then, (3) can be obtained:

4a $${\ddot{y}}_{1}+{\omega }_{1}^{2}{y}_{1}={ff}_{1}\left(t\right)$$

4b $${\ddot{y}}_{2}+{\omega }_{2}^{2}{y}_{2}={ff}_{2}\left(t\right)$$

where ${\omega }_{1}$ and ${\omega }_{2}$ represent the frequencies of mode 1 and mode 2, respectively. The force matrix and the relationship between {x} and {y} is

5a $$\left\{\begin{array}{c}{ff}_{1}\left(t\right)\\ {ff}_{2}\left(t\right)\end{array}\right\}={\left[s\right]}^{T}\left\{\begin{array}{c}\cos \left({\omega }_{n}t\right)\\ 0\end{array}\right\}=\frac{1}{\sqrt{2}m}\left\{\begin{array}{c}{f}_{1}\cos \left({\omega }_{d}t\right)\\ {f}_{2}\cos \left({\omega }_{d}t\right)\end{array}\right\}$$

5b $$\left\{\begin{array}{c}{x}_{1}\\ {x}_{2}\end{array}\right\}=\frac{1}{\sqrt{2}m}\left[\begin{array}{cc}1 & 1\\ 1 & -1\end{array}\right]\left\{\begin{array}{c}{Y}_{1}\cos \left({\omega }_{d}t\right)\\ {Y}_{2}\cos \left({\omega }_{d}t\right)\end{array}\right\}$$

### Decomposition for the weakly coupled resonators

The random fluctuations in an oscillator are referred to as noise and can be decomposed into amplitude and phase noise contributions. The ideal output of a weakly coupled resonator closed-loop circuit should be a perfect sinusoidal signal, with the phase $\varphi$ being constant and the mode frequency (${\omega }_{0}$) and amplitude (${V}_{0}$) varying as a function of the input tilt angle (${\theta }_{t}$)

6 $$v\left(t\right)={V}_{0}\left({\theta }_{t}\right)\sin \left[{\omega }_{0}\left({\theta }_{t}\right)t+\varphi \right]$$

**Fig. 3:**
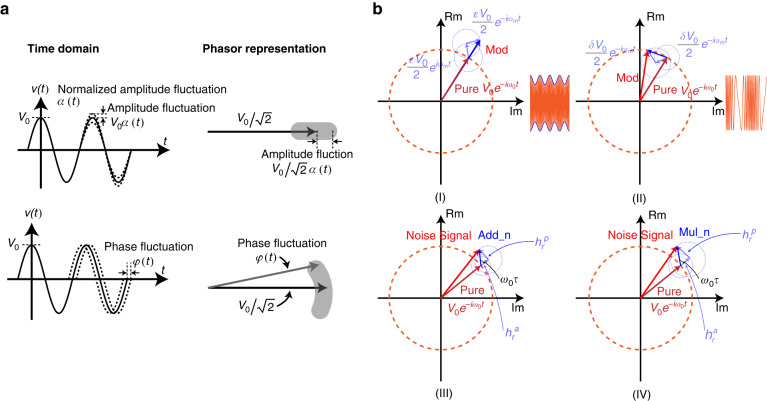
Schematic diagram of amplitude noise and phase noise, as well as amplitude noise modulation. **a** Schematic diagram of amplitude noise and phase noise in the time domain and phase domain. **b** Schematic diagram of amplitude noise modulation: the perturbation of an ideal signal by both additive and multiplicative noise in the phase domain. The ideal signal is a brown vector; the modulated signal and the noise signal are blue vectors; the output signal is a red vector, representing the vector summation of the ideal signal and the modulated signal (b)-I and (b)-II, and the vector summation of the ideal signal and the noise signal (b)-III and (d)-IV, given by the red vectors. ε is a small quantity related to noise. In the case of phase modulation (PM), the modulated signal has a constant phase shift of *π*/2 relative to the ideal signal

However, even when the input tilt angle is constant, the output signal of the mode-localized tilt sensor closed-loop circuit may still fluctuate in both amplitude and phase. These fluctuations can be characterized as amplitude and phase noise, as shown in Fig. 3a. The use of amplitude modulation and phase modulation also facilitates the presentation and superposition of noise and ideal signals, as illustrated in Fig. 3b. Figure 3b-I and b-II, respectively, show schematic diagrams of amplitude modulation and phase modulation in the phase domain, while Fig. 3b-III and b-IV, respectively, show schematic diagrams of the superposition of additive noise and multiplicative noise with the ideal signal in the phase domain. The difference between additive and multiplicative noises is in their respective perturbation vector lengths concerning *τ*. For additive noise, the length of the perturbation vector remains unaffected by *τ*, whereas for multiplicative noise, the length of the perturbation vector is dependent on *τ*.

**Fig. 4:**
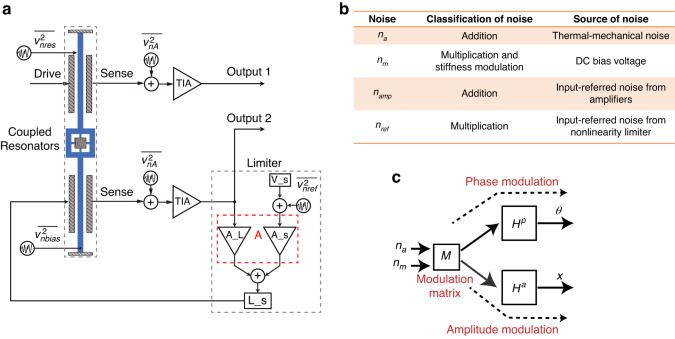
The main noise distribution in the weakly coupled resonator closed-loop circuit and decomposition of the phase and amplitude of the resonator. **a** The internal noise source mainly consists of mechanical thermal noise ($\bar{{v}_{{nres}}^{2}}$), and the external noise mainly consists of electronic noises: DC bias voltage noise, amplifier noise and noise for nonlinear circuits. **b** Classification for noises. **c** A schematic diagram of the modulation that decomposes the phase and amplitude of the resonator

The main noise sources in the weakly coupled resonator closed-loop circuit are illustrated in Fig. 4a. To comprehensively analyze the noise model, additive noise, multiplicative noise, and resonator stiffness noise modulation are considered. Additive noise directly affects the phase and amplitude noise, primarily originating from the mechanical thermal noise of the resonator. Multiplicative noise arises from the phase and amplitude noise resulting from gain fluctuations in the amplifiers. Resonator stiffness noise modulation results in changes in phase and amplitude noise due to DC bias voltage fluctuations and resonator nonlinearity. Figure 4b lists the classification and sources of noise involved in the weakly coupled resonator closed-loop circuit.

To facilitate the analysis of amplitude noise and phase noise in weakly coupled resonator closed-loop circuits, the weakly coupled resonator is regarded as two independent resonators based on the transformation matrix [s] (also referred to as modal equations). The motion equations are as follows:

7a $${\ddot{y}}_{1}+2{\xi }_{1}{\omega }_{1}{\dot{y}}_{1}+{\omega }_{1}^{2}{y}_{1}={ff}_{1}\left(t\right)$$

7b $${\ddot{y}}_{2}+2{\xi }_{2}{\omega }_{2}{\dot{y}}_{2}+{\omega }_{2}^{2}{y}_{2}={ff}_{2}\left(t\right)$$

where *ξ* is the damping ratio. By employing these modal equations, weakly coupled-resonator systems can be modeled as independent single resonators.

When the driving signal is superimposed with additive noise, the displacement response of the resonator is depicted in Fig. 3b-III, which can be decomposed into perturbations in both phase and amplitude. This decomposition is represented as^37^

8a $${h}_{a}^{p}\left(\tau ,t\right)={h}_{r}^{p}\left(\tau ,t\right)\frac{\sin \left({\omega }_{0}\tau \right)}{{V}_{0}}$$

8b $${h}_{a}^{a}\left(\tau ,t\right)={h}_{r}^{a}\left(\tau ,t\right)\cos \left({\omega }_{0}\tau \right)$$

In the above equation, ${h}_{r}^{p}\left(\tau ,t\right)$ and ${h}_{r}^{a}\left(\tau ,t\right)$ can be considered as the projections of ${h}_{{add}}$ onto the phase and amplitude directions of the ideal signal, as shown in Fig. 3b-III and b-IV.

The multiplicative noise can be considered to be the product of the impulse signal and the driving signal at time *τ*. Consequently, the displacement response of the resonator caused by the multiplicative noise is the convolution of the displacement response of the resonator due to the additive noise and the displacement response of the resonator with the ideal driving signal. Therefore, the phase and amplitude responses can be separately derived as

9a $${h}_{m}^{p}\left(\tau ,t\right)={h}_{r}^{p}\left(\tau ,t\right)\frac{\sin \left(2{\omega }_{0}\tau \right)}{2}$$

9b $${h}_{m}^{a}\left(\tau ,t\right)={h}_{r}^{a}\left(\tau ,t\right)\frac{{V}_{0}}{2}\left[1-\cos \left(2{\omega }_{0}\tau \right)\right]$$

The corresponding phase and amplitude responses caused by additive and multiplicative noise can be expressed in matrix form as

10 $$\left[\begin{array}{c}\theta \left(t\right)\\ x\left(t\right)\end{array}\right]=\int \left[\begin{array}{cc}{h}_{r}^{p} & \\ & {h}_{r}^{a}\end{array}\right]{M}^{T}\left(\tau \right){\left[\begin{array}{cc}{n}_{a}\left(\tau \right) & {n}_{m}\left(\tau \right)\end{array}\right]}^{T}d\tau$$

where *M* is the decomposition modulation matrix for additive and multiplicative noise

11 $$M\left(\tau \right)=\left[\begin{array}{cc}\frac{\sin \left({\omega }_{0}\tau \right)}{\frac{\gamma Q}{{k}_{{eff}}}} & \cos \left({\omega }_{0}\tau \right)\\ \frac{\sin \left(2{\omega }_{0}\tau \right)}{2} & \frac{\frac{\gamma Q}{{k}_{{eff}}}}{2}\left[1-\cos \left(2{\omega }_{0}\tau \right)\right]\end{array}\right]$$

Figure 4c shows the modulation process of the resonator for phase and amplitude responses induced by additive and multiplicative noise through the decomposition modulation matrix *M*.

### Nonlinearities of the weakly coupled resonators

These aforementioned analyses are based on the linear assumption of weakly coupled resonators. However, nonlinearities may occur in weakly coupled resonators and affect the performance of the mode-localized tilt sensor. Therefore, studying the nonlinear model contributes to understanding the impact of the nonlinear effect of weakly coupled resonators on the performance of the tilt sensor. The nonlinearity of weakly coupled resonators can be categorized into electrostatic driving nonlinearity and mechanical nonlinearity, wherein electrostatic driving nonlinearity includes capacitive current nonlinearity and capacitive force nonlinearity, while mechanical nonlinearity mainly is attributed to the nonlinear spring force^38,39^.

First, analyzing the noise resulting from the nonlinearity of the capacitive detection current. Based on the relationship between the driving voltage and the resonator’s displacement, the noise caused by the nonlinearity of the capacitive detection current is as follows^38^:

12 $${i}_{n}^{c}=2{\varGamma }_{c}{u}_{{ac}}{u}_{n},\,{\varGamma }_{c}=\frac{Q{\omega }_{0}{\eta }^{2}}{2k{V}_{0}}$$

where ${\varGamma }_{c}$ represents the noise conversion coefficient of the nonlinear effects in the capacitive detection current and $\eta$ is the electromechanical transduction coefficient.

Then, for the nonlinearity of the capacitive driving force. According to the relationship between the capacitive driving force and the resonator’s displacement. The noise caused by the nonlinearity of the capacitive driving force can be obtained as follows:

13 $${i}_{n}^{F}=2{\varGamma }_{F}{u}_{{ac}}{u}_{n},\,{\varGamma }_{F}\approx \frac{Q{\omega }_{0}{\eta }^{2}}{2k{V}_{0}}\left(1-j2\frac{Q\eta {V}_{0}}{{kd}}\right)$$

where ${\varGamma }_{F}$ is the noise transduction coefficient of the nonlinear effects in capacitive driving forces.

For the mechanical nonlinearity, the driving force caused by the voltage noise ${u}_{n}$ on the resonator is given by ${{F}_{n}=\eta u}_{n}$, which results in the resonator’s vibration amplitude ${x}_{n}=H\left(\omega \right){F}_{n}\approx \frac{{\eta u}_{n}}{{k}_{{eff}}}$, where $H\left(\omega \right)$ is the transfer function of the resonator. The driving force caused by the mechanical nonlinearity spring hardening effect on the resonator is given by ${F}_{n}^{k}=2{k}_{{eff}}{k}_{3}{x}_{0}{x}_{n}$. The current noise generated due to the mechanical nonlinearity spring hardening effect is as follows:

14 $${i}_{n}^{k}=2{\varGamma }_{k}{u}_{{ac}}{u}_{n},\,{\varGamma }_{k}=j\frac{3Q{\omega }_{0}{\eta }^{4}{V}_{0}}{2{d}^{2}{k}^{3}}$$

where ${\varGamma }_{k}$ is the noise transduction coefficient of the mechanical nonlinearity spring hardening effect.

### The DD noise analysis model based on a weakly coupled resonators close-loop circuit

**Fig. 5:**
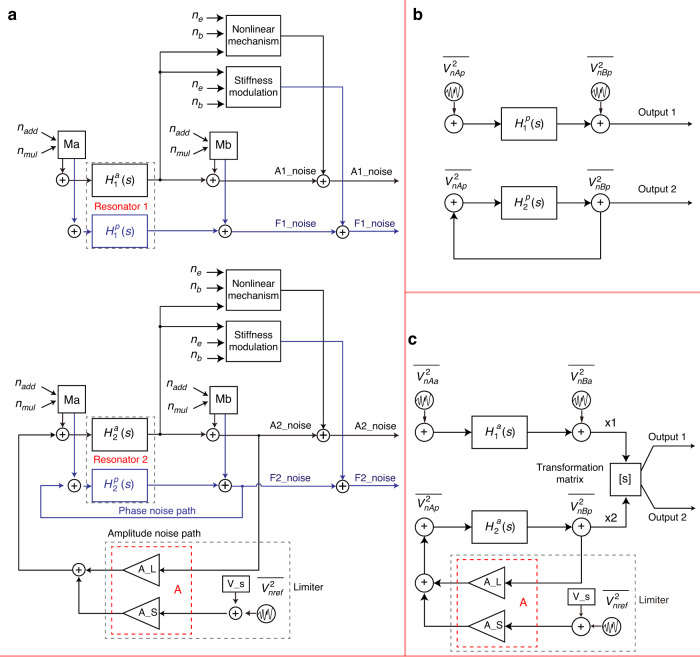
Phase and amplitude noise channels in the weakly coupled resonator closed-loop circuit. **a** The blue part is the phase noise channel, and the black part is the amplitude noise channel. Resonator 2 is in the closed-loop oscillating circuit, and the main nodes include A, B, and C. Node A is the noise source of the resonator driving node, node B is the noise source of the resonator detecting node, and node C is the reference noise source of the nonlinear limiting circuit. Resonator 1 is open-loop and only includes nodes A and B. **b** Phase noise path in the weakly coupled resonator closed-loop circuit. **c** Amplitude noise path in the weakly coupled resonator closed-loop circuit

Based on the transformation matrix, the weakly coupled resonators can be analyzed as two independent resonators for noise decomposition, where each resonator system contains both a phase noise channel and an amplitude noise channel. As shown in Fig. 5, the blue part represents the phase noise channel, while the black part represents the amplitude noise channel. When the weakly coupled resonators closed-loop circuit stabilizes, the vibration displacements of resonator 1 and resonator 2 become fixed due to the nonlinear limiting circuit and the modal localization effect. For example, the vibration displacement of resonator 2 stabilizes at ${x}_{20}$ because of the presence of the nonlinear limiting circuit, while the vibration displacement of resonator 1 stabilizes at ${x}_{10}$ due to the modal localization effect.

First, resonator 2 in the closed-loop circuit is analyzed. There are three main noise nodes in the loop, namely A, B, and C. The noise in each node is divided into additive noise and multiplicative noise, which are applied to the weakly coupled resonator closed-loop circuit through the corresponding noise modulation matrix. Node A is the noise source for driving the resonator, which mainly includes the thermal-mechanical noise of the resonator and the DC bias voltage noise. Node B is the noise source for detecting the resonator, which includes amplifier noise and DC bias voltage noise. Node C is the reference noise source of the nonlinear limiting circuit. Because the parasitic effects of the nonlinear limiting circuit can also be equivalent to a loop filter, the nonlinear limiting circuit is only regarded as being sensitive to amplitude noise and insensitive to phase noise. Therefore, the noise modulation matrix of node C is defined as $${M}_{C}=\left[\begin{array}{cc}0 & 1\\ 0 & A\end{array}\right]$$.

Figure 4a shows the phase noise channel of the weakly coupled resonators closed-loop circuit through the demodulated resonator 1 and resonator 2. Resonator 1 is in the open-loop path, while resonator 2 is in the closed-loop path. At node A, there is additive noise: resonator mechanical thermal noise ${n}_{m}$ and multiplicative noise bias voltage noise ${n}_{b}$. Based on to (10), the phase noise at node A of resonator 1 and resonator 2 can be obtained as follows:

15 $${n}_{A}^{p}=\left[\begin{array}{cc}{n}_{m} & {n}_{b}\end{array}\right]{M}_{A}\left[\begin{array}{c}1\\ 0\end{array}\right]=\frac{\gamma Q}{{k}_{{eff}}}\frac{1}{{x}_{0}}\cos \left({\omega }_{0}t\right){n}_{m}+\frac{1}{2}\sin \left(2{\omega }_{0}t\right){n}_{b}$$

Based on to these node noise and combining the nonlinear effect caused noise, the amplitude noise can be obtained as

16 $$\left[\begin{array}{c}{S}_{{AM}1}\left(\Delta \omega \right)\\ {S}_{{AM}2}\left(\Delta \omega \right)\end{array}\right]=\frac{1}{\sqrt{2}m}\left[\begin{array}{c}{S}_{{AM}1}^{{linear},0}+{S}_{{AM}1}^{{linear},1/{f}^{2}}\frac{1}{{\Delta \omega }^{2}}+{\left|\varGamma \right|}^{2}{R}_{m}^{2}{u}_{{ac}}^{2}{\left|{u}_{n,1/f}\right|}^{2}{\left(\frac{{\omega }_{c}}{\Delta \omega }\right)}^{2}\\ {S}_{{AM}2}^{{linear},0}+{S}_{{AM}2}^{{linear},1/{f}^{2}}\frac{1}{{\Delta \omega }^{2}}+{\left|\varGamma \right|}^{2}{R}_{m}^{2}{u}_{{ac}}^{2}{\left|{u}_{n,1/f}\right|}^{2}{\left(\frac{{\omega }_{c}}{\Delta \omega }\right)}^{2}\end{array}\right]$$

where ${S}_{{AM}1,{AM}2}^{{linear},0}$ and ${S}_{{AM}1,{AM}2}^{{linear},1/{f}^{2}}$ represent the amplitude white noise and amplitude $1/{f}^{2}$ noise, respectively; further details are provided in the Supplementary Material. From (16), the optimal working point for the amplitude of a weakly coupled resonator with nonlinear noise is derived as

17 $${x}_{0}=\sqrt{\left(1+\sqrt{1+{\left(2\frac{{Q}^{2}\eta \gamma }{{k}_{{eff}}^{2}}\right)}^{2}}\right){\Bigg{/}}\frac{3Q{\eta }^{2}}{{d}^{2}{k}_{{eff}}^{2}}}$$

### Numerical simulation and verification of the DD noise analysis model

To validate the aforementioned theoretical analysis, a system model for simulating the weakly coupled resonator’s closed-loop circuit is established using MATLAB/Simulink, as shown in Supplementary Material Fig. S2.

**Fig. 6:**
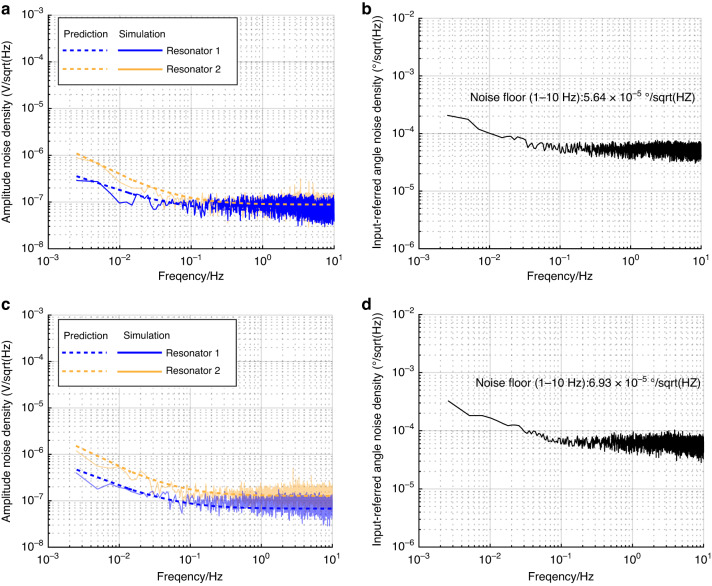
The results of the model simulation and prediction. The consistency between the system model simulation and the system decomposition model amplitude noise prediction for linear in **a** and nonlinear coupled resonators in **c**. The input-referred angle noise density on the tilt sensor for linear in **b** and nonlinear coupled resonators in **d**

In the simulation of both linear and nonlinear coupled resonators, the third-order spring coefficient ${k}_{2}$ is set to zero and $5.05\times {10}^{7}N/{m}^{3}$, respectively. Figure 6a, c demonstrate the comparisons between the theoretical and simulation results of the weakly coupled resonators closed-loop circuit system noise model. Figure 6b, d show the input-referred angular noise density, determined by calculating the AR sensitivity from the experimental results, resulting in values of $5.64\times {10}^{-5}{\rm{^\circ }}/\sqrt{{Hz}}$ and $6.93\times {10}^{-5}{\rm{^\circ }}/\sqrt{{Hz}}$, respectively. The consistency between the system model simulation and the system decomposition model’s amplitude noise prediction verifies the model’s accuracy in describing both the linear and nonlinear resonator regions.

**Fig. 7:**
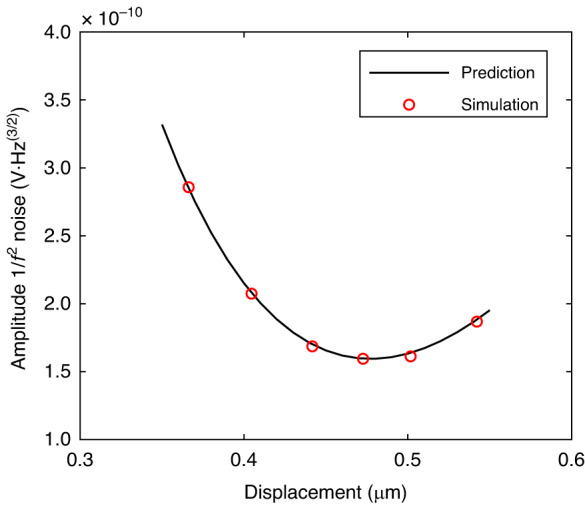
Comparison of theoretically predicted amplitude ${\boldsymbol{1}}/{{\boldsymbol{f}}}^{{\boldsymbol{2}}}$ noise and simulation results. The predictable results are calculated from (17), and the simulation results are simulated from the system simulation model using MATLAB/Simulink

Figure 6 shows that the nonlinear effect will increase the amplitude $1/{f}^{2}$ noise due to nonlinearity. On the other hand, based on (17), there is an optimal nonlinear working point for amplitude $1/{f}^{2}$ noise. The simulation results and theoretical model predictions are in good agreement, and there is an optimal nonlinear working point for amplitude $1/{f}^{2}$ noise from Fig. 7. This conclusion is used as a reference for the optimized design of low-frequency amplitude $1/{f}^{2}$ noise in the mode-localized tilt sensor.

### The performance of the mode-localized tilt sensor

**Fig. 8:**
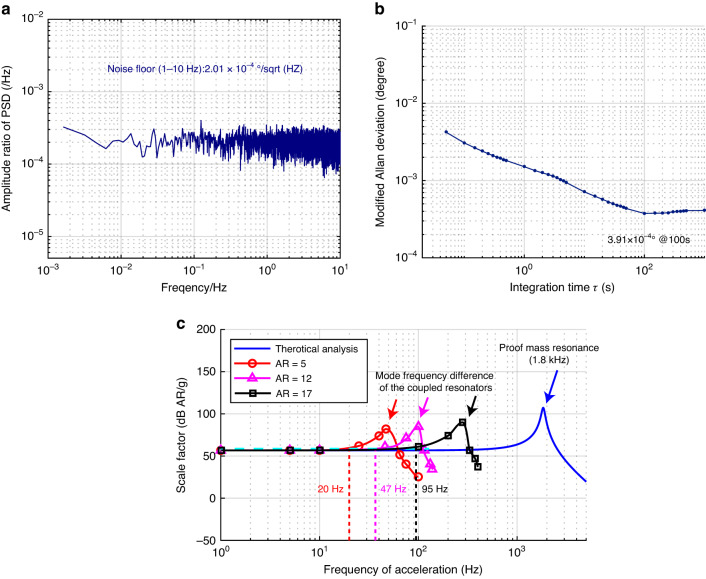
Performance of the mode-localized tilt sensor. The input-referred angle noise density in **a** and the modified Allan deviation in **b** for the mode-localized tilt sensor. **c** Bandwidth of the mode-localized tilt sensor under different ARs

Utilizing the DD noise analysis model to improve the amplitude $1/{f}^{2}$ noise, the optimal operating point exhibits better low-frequency noise performance. In our previous work^26^, the optimal operating point for white noise of the mode-localized tilt sensor is determined by the amplitude ratio as approximately 1.22. The amplitude ratios of the PSD and modified Allan deviation are calculated, and the results are shown in Fig. 8a, b. The input-referred amplitude ratio of PSD for the tilt sensor is $2.01\times {10}^{-4}{\rm{^\circ }}/\sqrt{{Hz}}$, and the modified Allan deviation is $3.91\times {10}^{-4}{\rm{^\circ }}$ when the drive voltage signal is 18 ${mV}$(as determined by theoretical analysis) and the amplitude ratio is 1.22. The noise level of the experimental result is larger than that of the simulation result because of the impacts from board-level circuit parasitic capacitances and the drive signal noise. The simulation result of the amplitude $1/{f}^{2}$ noise coefficient on the tilt sensor for nonlinearity is 0.0022, as calculated from Fig. 6d, and the experimental result of the amplitude $1/{f}^{2}$ noise coefficient is 0.0157, as calculated from Fig. 8a. The experimental results are smaller than the simulation results because the amplitude $1/{f}^{2}$ noise closely approximates white noise.

The resonance frequency and quality factor of the proof mass are 1.8${kHz}$ and 927, respectively, which shows that the maximum bandwidth limit on the tilt sensor is determined by the characterization of the proof mass. The amplitude ratios of the weakly coupled resonators are set to 5, 12, and 17, which are used to investigate the bandwidth of the tilt sensor at different amplitude ratios, as shown in Fig. 8c. The effective bandwidths of the tilt sensor are 20${Hz}$, 47${Hz}$, and 95${Hz}$ when the amplitude ratios are 5, 12, and 17, respectively. Increasing the amplitude ratio increases the effective bandwidth. Peaks occur when the drive frequency of the tilt sensor is at the mode frequency difference of the weakly coupled resonators, which also affects the effective bandwidth for the tilt sensor.

## Discussion and conclusion

In this paper, a DD noise analysis model based on a weakly coupled resonator closed-loop circuit is established to optimize the noise performance of mode-localized tilt sensors. The DD noise model separates weakly coupled resonators using the decoupling method considering the nonlinearity of these resonators. By integrating the decoupled weakly coupled resonators, the model decomposes the weakly coupled resonator closed-loop circuit into paths for amplitude noise and phase noise analysis, realizing an expression of system noise in the weakly coupled resonator closed-loop circuit. Simulation verification is conducted using MATLAB/Simulink and demonstrates that the model has a high accuracy when compared with theoretical analysis. The mode-localized tilt sensor works at the optimal operating point, an input-referred instability of $3.91\times {10}^{-4}{\rm{^\circ }}$, where an input-referred amplitude ratio of PSD of $2.01\times {10}^{-4}{\rm{^\circ }}/\sqrt{{Hz}}$ is obtained. In addition, an effective bandwidth of 47${Hz}$ is also measured on the mode-localized tilt sensor. The DD noise analysis model for weakly coupled resonators demonstrates potential in utilizing the mode-localized paradigm for optimizing various sensors, including accelerometers, electrometers, and mass sensors. Future work on enhancing the resolution of mode-localized sensors could involve the integration of ultralow mechanical coupling structures, optimal operating Duffing nonlinearity, and ultraprecise level bias voltages for different applications.

### Supplementary information

Supplementary Materials
