# Supplementary material for: A decouple-decomposition noise analysis model for closed-loop mode-localized tilt sensors
Source: Microsyst Nanoeng. 2023 Dec 21;9:157. doi: 10.1038/s41378-023-00614-z (PMC10733308; doi:10.1038/s41378-023-00614-z)
Supplement: Supplementary file 1 — Supplementary Materials [file 41378_2023_614_MOESM1_ESM.docx]

#### Supplementary Materials

#### A decouple-decomposition noise analysis model for closed-loop mode-localized tilt sensors

*Kunfeng Wang^a,b,c^, XingYin Xiong^a^, Zheng Wang^d^, Liangbo Ma^a,b^, BoWen Wang^a,b^, WuHao Yang^a^, Xiaorui Bie^a^, ZhiTian Li^a^, XuDong Zou^a,b,d,*^*

^a^ The State Key Laboratory of Transducer Technology, Aerospace Information Research Institute, Chinese Academy of Sciences, Beijing, CHINA

^b^ School of Electronic, Electrical and Communication Engineering, University of Chinese Academy of Sciences, Beijing, CHINA

^c^ Ming Hsieh Department of Electrical and Computer Engineering, University of Southern California, Los Angeles, USA

^d^ Shangdong Key Laboratory of Low-altitude Airspace Surveillance Network Technology, QiLu Aerospace Information Research Institute, Jinan, CHINA

**The detail of deviation for DD noise analysis model**

The phase noise at node A of resonator 1 and resonator 2 can be obtained as follows:

$n_{A}^{p}=\left[ \begin{matrix} n_{m} & n_{b} \end{matrix} \right]M_{A}\left[ \begin{matrix} 1 \\ 0 \end{matrix} \right]=\frac{\gamma Q}{k_{eff}}\frac{1}{x_{0}}\cos\left( \omega_{0}t \right)n_{m}+\frac{1}{2}\sin\left( 2\omega_{0}t \right)n_{b}$ (S1)

From the above equation, it can be seen that the thermal-mechanical noise $n_{m}$ of the resonator has an effect on the first harmonic phase noise of the resonator at node A, while the bias voltage noise $n_{b}$ at node A only affects the second harmonic phase noise of the resonator. Therefore, the effect of the bias voltage noise $n_{b}$ at node A on the phase noise can be ignored.

$S_{A}^{P}\left( \Delta\omega\right)=\left( \frac{\gamma Q}{k_{eff}}\frac{1}{x_{0}} \right)^{2}\bar{n_{m}^{2}}$ (S2)

Similarly, at node B, according to the phase transfer function $H^{p}\left( s \right)=\frac{{\omega_{0}}/{2Q}}{s+{\omega_{0}}/{2Q}}=\frac{\omega_{c}}{s+\omega_{c}}$, it can be derived that the phase noise at node B caused by the amplifier noise $n_{amp}$ is given by

$S_{B}^{P}\left( \Delta\omega\right)=\left( \frac{1}{x_{0}} \right)^{2}\left( \bar{n_{amp}^{2}}+\bar{n_{b}^{2}} \right)$ (S3)

The phase noise for resonator 1 and resonator 2 can be respectively obtained

$S_{r1}^{P}\left( \Delta\omega\right)=\left( \frac{\gamma Q}{k_{eff}}\frac{1}{x_{0}} \right)^{2}\bar{n_{m}^{2}}\omega_{c}^{2}+\left( \frac{1}{x_{0}} \right)^{2}\frac{\bar{n_{amp}^{2}}+\bar{n_{b}^{2}}}{{\Delta\omega}^{2}}$ (S4a)

$S_{r2}^{P}\left( \Delta\omega\right)=\left[ \left( \frac{\gamma Q}{k_{eff}}\frac{1}{x_{0}} \right)^{2}\bar{n_{m}^{2}}+\bar{n_{amp}^{2}} \right]\omega_{c}^{2}+\left( \frac{1}{x_{0}} \right)^{2}\frac{\bar{n_{amp}^{2}}+\bar{n_{b}^{2}}}{{\Delta\omega}^{2}}$ (S4b)

Fig. 4 (b) shows the amplitude noise channel for resonator 1 and resonator 2 of the weakly coupled resonator closed-loop circuit through demodulation. At node A, there exists an additive noise $n_{m}$ (resonator mechanical thermal noise) and a multiplicative noise $n_{b}$ (bias voltage noise). According to (S1), the amplitude noise at node A for resonator 1 and resonator 2 can be obtained

$n_{A}^{a}=\left[ \begin{matrix} n_{m} & n_{b} \end{matrix} \right]M_{A}\left[ \begin{matrix} 0 \\ 1 \end{matrix} \right]=n_{m}\cos\left( \omega_{0}t \right)+\frac{x_{0}}{2\frac{\gamma Q}{k_{eff}}}n_{b}$ (S5)

The amplitude noise spectral density at node A is given by

$S_{A}^{a}\left( \Delta\omega\right)=\bar{n_{m}^{2}}+\left( \frac{x_{0}}{2\frac{\varrho Q}{k_{eff}}} \right)^{2}\frac{\bar{n_{b}^{2}}}{{\Delta\omega}^{2}}$ (S6)

Similarly, the amplitude noise spectral density at node B and node C can be respectively calculated

$S_{B}^{a}\left( \Delta\omega\right)=\bar{n_{B}^{2}}+\left( \frac{x_{0}}{2} \right)^{2}\frac{\bar{n_{b}^{2}}}{{\Delta\omega}^{2}}$ (S7)

$S_{C}^{a}\left( \Delta\omega\right)=\bar{n_{ref,m}^{2}}+\frac{\bar{n_{ref,1/f}^{2}}}{{\Delta\omega}^{2}}$ (S8)

where $\bar{n_{B}^{2}}$ represents the noise at node B

$\bar{n_{B}^{2}}=\frac{\bar{n_{ref,m}^{2}}}{A}+\bar{n_{amp}^{2}}F^{2}+\bar{n_{ref,m}^{2}}$, $F^{2}=1+\frac{R_{x}\omega_{0}C_{in}}{A}$ (S9)

where $C_{in}$ is the input capacitance. Considering the amplitude noise near the resonance frequency, the amplitude noise at node A is suppressed in the forward direction by the resonator and limited in the reverse direction by the limiter (which can be equivalent to a low-pass filter). Therefore, the amplitude noise at node A can be neglected, while the amplitude noise at nodes B and C directly affects the output signal. Besides, considering the nonlinearities of the weakly coupled resonators result in up-conversion noise entering the loop and being amplified in the feedback circuit. The output amplitude noise spectral density can be expressed as

$S_{nonlinear}^{a}\left( \Delta\omega\right)=\left| \Gamma\right|^{2}R_{m}^{2}u_{ac}^{2}\left| u_{n,1/f} \right|^{2}\left( \frac{\omega_{c}}{\Delta\omega} \right)^{2}$ (S10)

where $u_{n,1/f}$ is the flicker noise at the nodes, $\Gamma=\left| \Gamma_{c} \right|+\left| \Gamma_{F} \right|+\left| \Gamma_{k} \right|$ is the noise transduction coefficient of nonlinear effects^1^. The (S7), (S8) and (S10) into the decomposition relationship of the (5), then combining the output amplitude noise (S10), the amplitude noise can be obtained as

$\left[ \begin{matrix} S_{AM1}\left( \Delta\omega\right) \\ S_{AM2}\left( \Delta\omega\right) \end{matrix} \right]=\frac{1}{\sqrt{2}m}\left[ \begin{matrix} S_{AM1}^{linear,0}+S_{AM1}^{linear,1/{f^{2}}}\frac{1}{{\Delta\omega}^{2}}+\left| \Gamma\right|^{2}R_{m}^{2}u_{ac}^{2}\left| u_{n,1/f} \right|^{2}\left( \frac{\omega_{c}}{\Delta\omega} \right)^{2} \\ S_{AM2}^{linear,0}+S_{AM2}^{linear,1/{f^{2}}}\frac{1}{{\Delta\omega}^{2}}+\left| \Gamma\right|^{2}R_{m}^{2}u_{ac}^{2}\left| u_{n,1/f} \right|^{2}\left( \frac{\omega_{c}}{\Delta\omega} \right)^{2} \end{matrix} \right]$ (S11)

where $S_{AM1,AM2}^{linear,0}$ and $S_{AM1,AM2}^{linear,1/{f^{2}}}$ represent the amplitude white noise and amplitude $1/{f^{2}}$ noise, respectively.

$\left[ \begin{matrix} S_{AM1}^{linear} \\ S_{AM2}^{linear} \end{matrix} \right]=\frac{1}{\sqrt{2}m}\left[ \begin{matrix} \Xi+\left[ \frac{x_{0}^{2}}{4}\bar{n_{b}^{2}}\left( 2+\omega_{c}^{2} \right)+\left( \frac{\gamma Q}{k_{eff}} \right)^{2}\omega_{c}^{2}\bar{n_{ref,1/f}^{2}} \right]\frac{1}{{\Delta\omega}^{2}} \\ \Xi+\left[ \left( \frac{\gamma Q}{k_{eff}} \right)^{2}\omega_{c}^{2}\bar{n_{ref,1/f}^{2}}+\frac{x_{0}^{2}}{4}\bar{n_{b}^{2}}\omega_{c}^{2} \right]\frac{1}{{\Delta\omega}^{2}} \end{matrix} \right]$ (S12)

where $\Xi=\left( {\gamma Q}/{k_{eff}} \right)^{2}\omega_{c}^{2}\left( \bar{n_{m}^{2}}\pm\bar{n_{ref,m}^{2}} \right)+\bar{n_{amp}^{2}}\left( {1\pm F}^{2} \right)$.

In the (S10), the noise transduction coefficient of nonlinear effects $\Gamma$ is expressed as

$\left| \Gamma\right|=\left| \Gamma_{c} \right|+\left| \Gamma_{F} \right|+\left| \Gamma_{k} \right|=\frac{Q\omega_{0}\eta^{2}}{2k_{eff}^{2}}\frac{\varrho Q}{x_{0}}+\frac{Q\omega_{0}\eta^{2}}{2k_{eff}^{2}}\frac{\varrho Q}{x_{0}}\sqrt{1+\left( 2\frac{Q^{2}\eta\varrho}{k_{eff}^{2}} \right)^{2}}+\frac{3Q^{2}\omega_{0}\eta^{4}\varrho}{2d^{2}k_{eff}^{4}}x_{0}$ (S13)

The optimal working point for the amplitude of a weakly coupled resonator with nonlinear noise can be obtained from the (S13).

$x_{0}=\sqrt{\left( 1+\sqrt{1+\left( 2\frac{Q^{2}\eta\gamma}{k_{eff}^{2}} \right)^{2}} \right)/{\frac{3Q\eta^{2}}{d^{2}k_{eff}^{2}}}}$ (S14)

From the S(14), we can obtain that when the amplitude of a weakly coupled resonator is smaller than the $x_{0, optimal}$ the nonlinearity of the capacitive driving force $\left| \Gamma_{F} \right|$ and the nonlinearity of the capacitive detection current $\left| \Gamma_{c} \right|$ are dominant the amplitude $1/f^{2}$ noises, while exceed the $x_{0, optimal}$ the mechanical nonlinearity is dominant the amplitude $1/f^{2}$ noises.

**Table I.** **Dimensions of the mode-localized tilt sensor.**


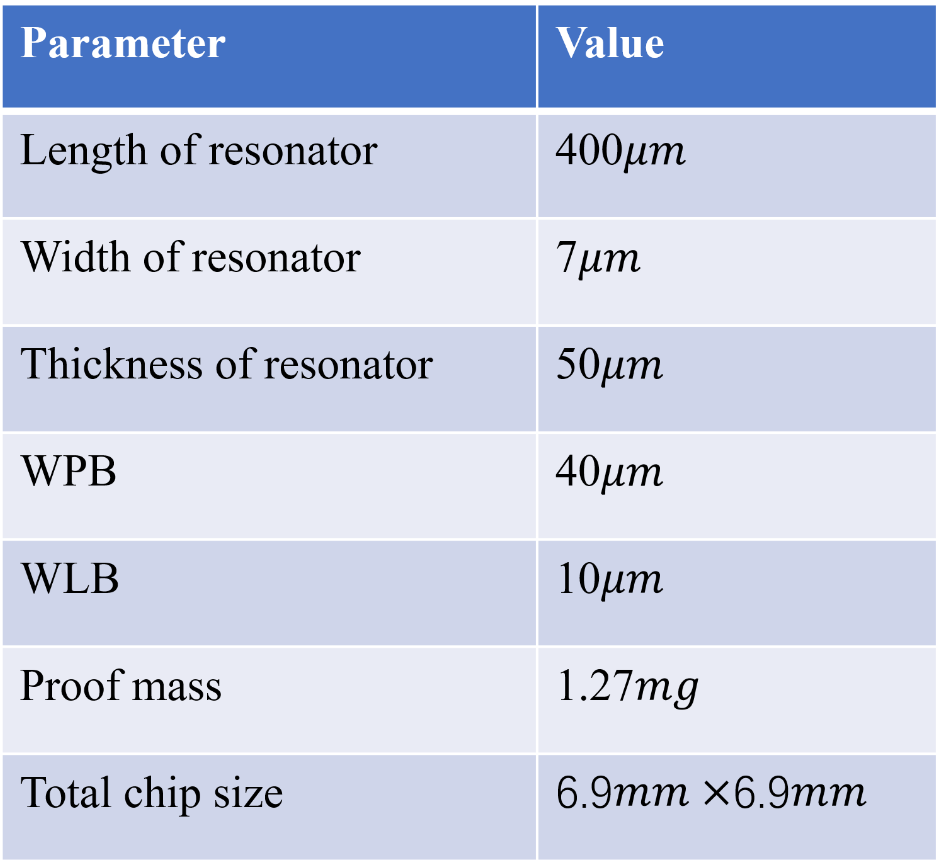


**Table II. Parameters of the simulation model.**


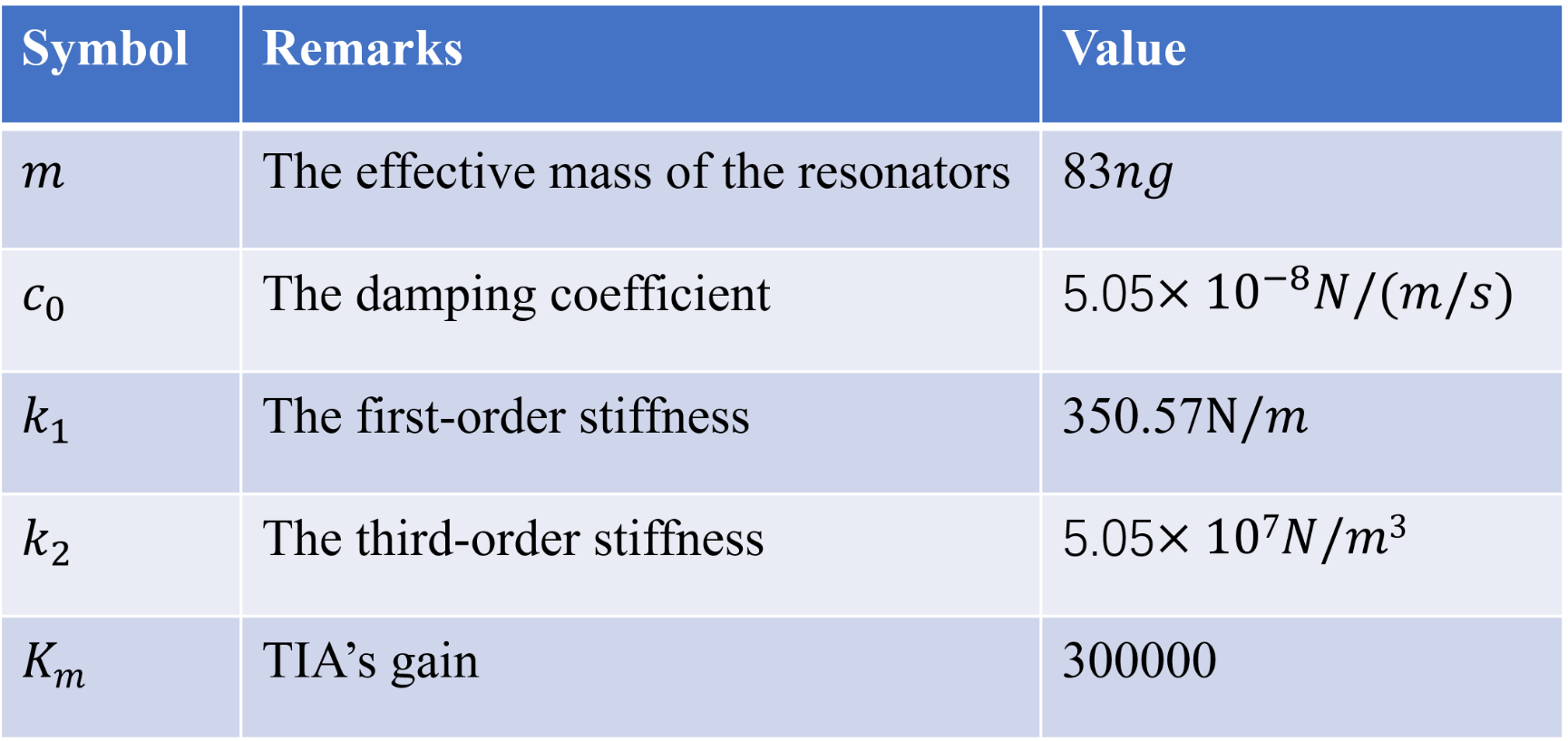


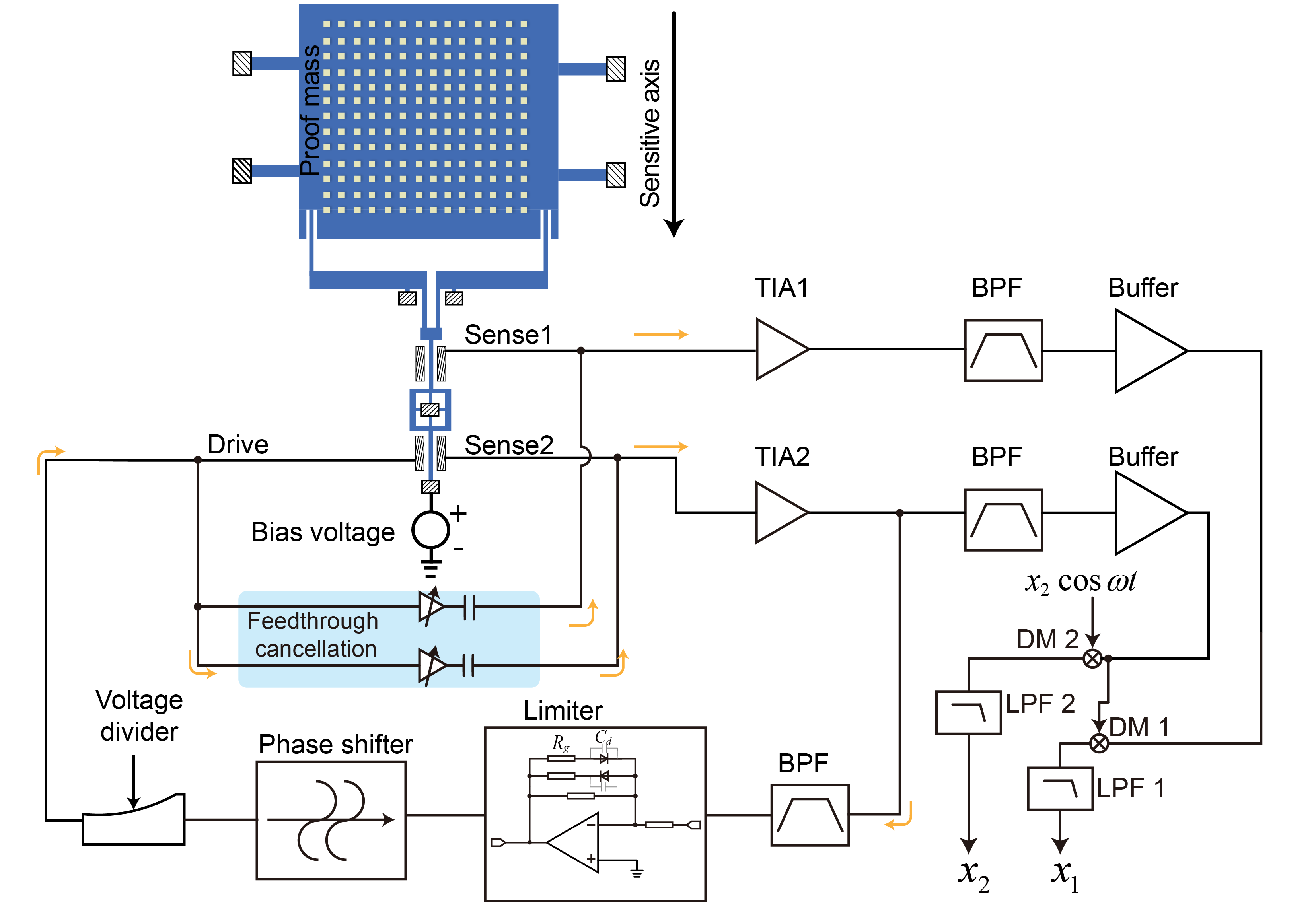


Fig. S1 The experimental configuration. The TIA is trans-impedance amplifiers, BPF is the bandpass filter, LPF is the low pass filter, and the DM is the demodulator.


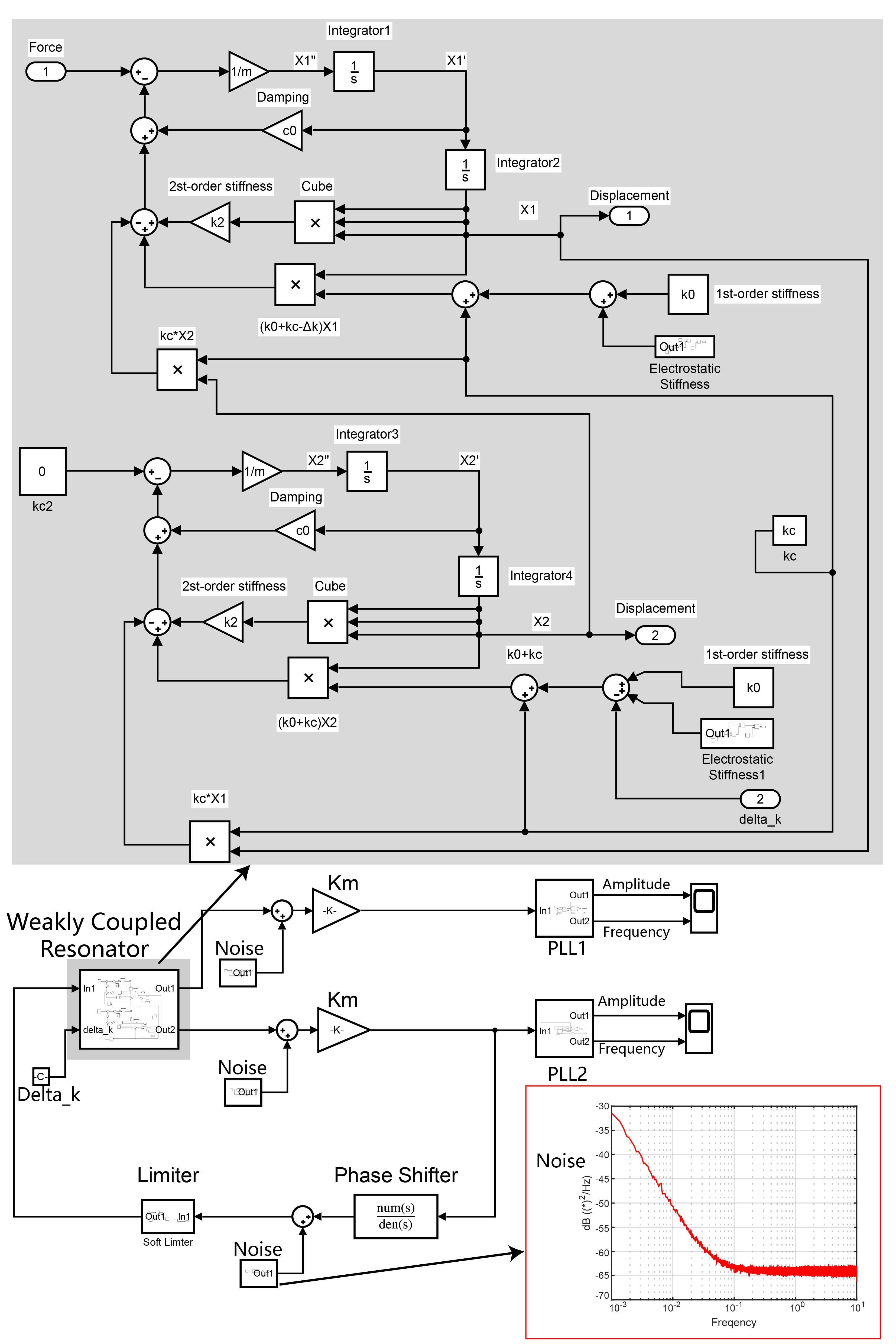


Fig. S2 The noise simulation model of the weakly coupled resonant oscillator system. The system noise model includes: weakly coupled resonator model, front-end amplifier, phase shifter, limiter, noise sources at various nodes (inset figure), and phase-locked loops (PLL) module for measuring frequency and amplitude. The parameters of the weakly coupled resonator and related circuit parameters are summarized at Table I and Table II.


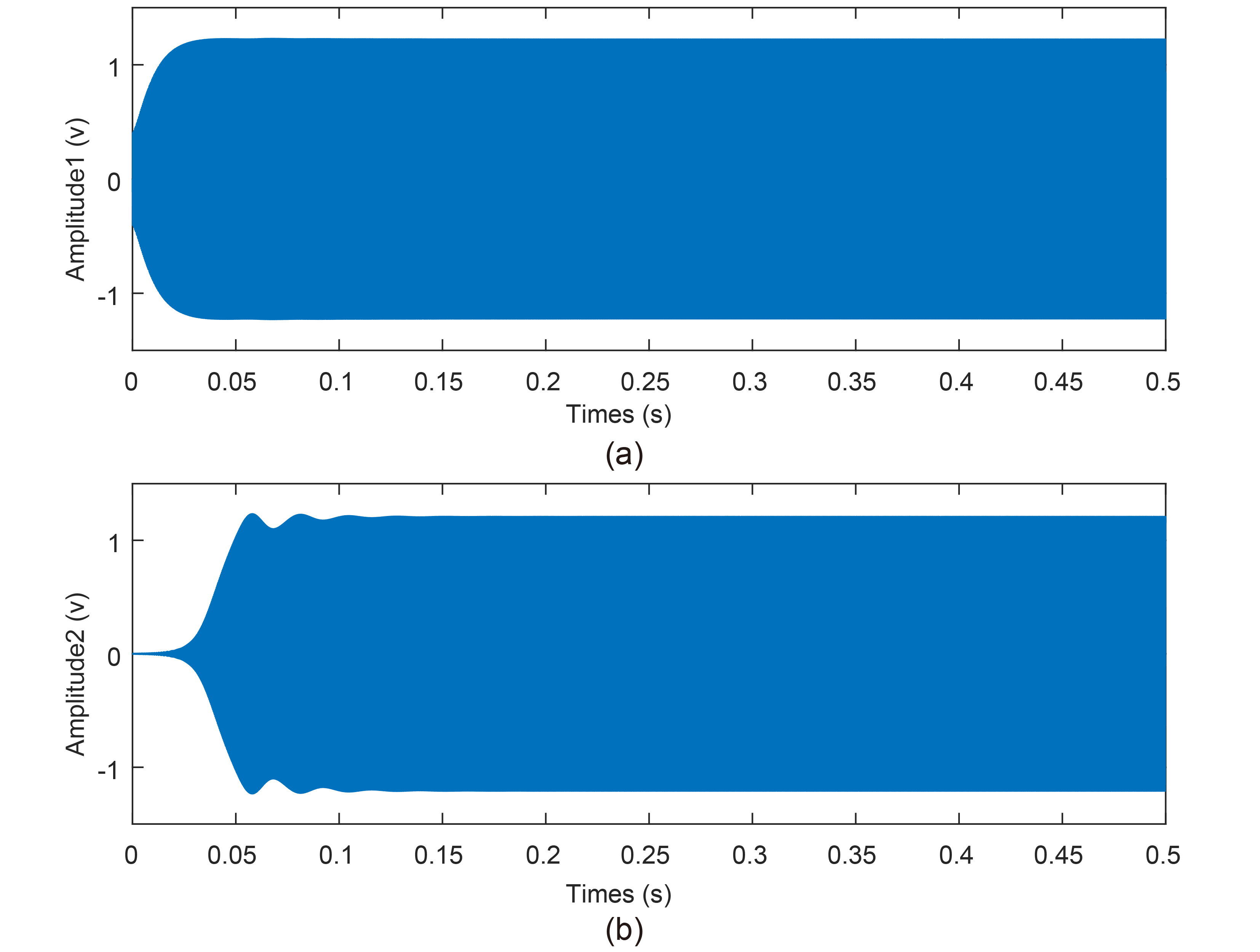


Fig. S3 Output waveforms of simulation model of the weakly coupled resonant oscillator system without noise, resonator 1 outputs at (a) and resonator 2 outputs at (b).

**References:**

1 Kaajakari, V., Koskinen, J. K. & Mattila, T. Phase noise in capacitively coupled micromechanical oscillators. *IEEE Transactions on Ultrasonics, Ferroelectrics, and Frequency Control* **52**, 2322-2331 (2005). <https://doi.org:10.1109/TUFFC.2005.1563277>
